# Angular stable plate fixation provides favorable biomechanical stability in simulated T-shaped acetabular fractures: a biomechanical study

**DOI:** 10.2340/17453674.2024.42490

**Published:** 2024-11-28

**Authors:** Moritz F LODDE, Christoph KATTHAGEN, Matthias KLIMEK, Karl ABSHAGEN, Christian PEEZ, Arian GROßE-ALLERMANN, Michael J RASCHKE, Oliver RIESENBECK

**Affiliations:** Department of Trauma, Hand and Reconstructive Surgery, University Hospital Münster, Münster, Germany

## Abstract

**Background and purpose:**

The treatment of acetabular fractures remains technically demanding. In the case of reduced bone quality or fracture morphology reducing the amount of bone available for fixation, locking plates should provide considerable advantages. The aim of the present study was to compare conventional and locking plate fixation. It was hypothesized that locking plate fixation provides less displacement and higher construct stiffness.

**Methods:**

A T-shaped acetabular fracture was simulated in 16 synthetic pelvic models. The fracture was addressed with a biplanar 10-hole 2-column plate buttressing the medial acetabular wall. Optical markers were attached to the fracture sites for motion tracking. Standardization of the acetabulum loading mechanism was performed using a unipolar hemiarthroplasty. The primary outcome measure was displacement at the fracture sites. The secondary outcome measure was the construct stiffness (N/mm).

**Results:**

Fracture displacement was less in the group of angular stable implants compared with the group fixed with conventional non-locking implants. Under cyclic loading displacement was less in the group of locking plate fixation. No differences in mean initial axial stiffness were detected between locking plate fixation (407 N/mm) and conventional plating (308 N/mm, ∆ 99 N/mm, 95% confidence interval –48 to 245).

**Conclusion:**

We showed that locking plate fixation buttressing the medial acetabular wall achieved less fracture displacement but showed no differences in axial stiffness compared with conventional plating.

The incidence of acetabular fractures in patients aged ≥ 60 years increased substantially in the last decades and is reported to be the most rapidly growing segment of acetabular trauma [1]. In particular, the entities of anterior column and quadrilateral plate fractures are observed [1]. These fractures are associated with an increasing prevalence of osteoporosis amongst an aging population [1,2]. Acetabular fractures among elderly patients with involvement of the medial wall and combined with reduced bone quality are challenging to treat. Laflamme et al. described that internal fixation buttressing the quadrilateral plate is a feasible alternative to total hip arthroplasty [2]. Results of a case series including 62 elderly patients showed that open reduction and internal fixation of acetabular fractures is safe and reliable [3]. However, significant loss of reduction was associated with reduced bone quality [2].

It is generally accepted that conventional plating has a higher failure rate in poor bone stock compared with locked plating. In the case of reduced bone quality and complex fracture morphology, locking plates should provide considerable advantage. An additional main indication for using angular stability is fractures close to a joint. The potentially inferior outcomes in the elderly patient led to specific treatment pathways, especially the combination of internal fixation and arthroplasty. This surgical approach allows early full weightbearing [4]. However, it is associated with added technical complexity due to the often unstable fracture limiting the implant positioning and with a potentially significant perioperative risk following total hip arthroplasty in the elderly patient group [4-6]. A feasible alternative might be the use of a locking implant with angular stable screws.

The aim of the present biomechanical study was to compare conventional plate fixation with locking plate fixation, both buttressing the quadrilateral plate in a simulated T-shaped acetabular fracture. It was hypothesized that the locking plates are biomechanically superior with increased stability, a higher construct stiffness, and less fracture displacement.

## Methods

**Figure 1:**
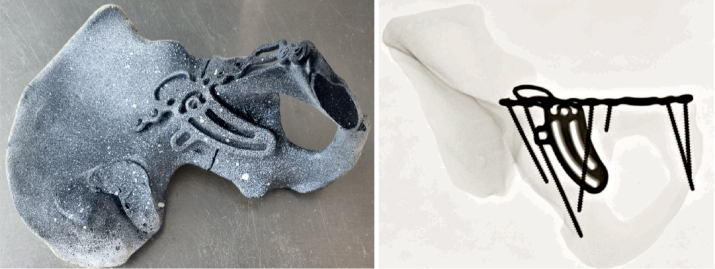
A T-shaped acetabular fracture was fixed with a biplanar 10-hole 2-column plate osteosynthesis using either conventional screws or angular stable screws. On the left is a specimen after anatomic repositioning and fixed plate osteosynthesis. The specimens were sprayed for optical motion tracking. On the right the corresponding radiograph is displayed.

A T-shaped acetabular fracture according to Judet and Letournel’s classification system was created in 16 synthetic pelvic models (Model #LS4060, Synbone, Zizers, Switzerland; Figure 1). The model was made of solid foam and dense cancellous bone lacquered with 2K brilliant varnish.

### Specimen preparation

The T-shaped acetabular fractures were simulated via osteotomies of the os ilium, os ischium, and os pubis. Osteotomies were set using custom-made saw cut templates. The pelvises were assigned to 2 groups (Group A conventional plate fixation, Group B locking plate fixation) of 8 specimens each. After anatomic reduction, the fracture was addressed with a biplanar 10-hole 2-column plate (ITS, PRS Pelvic Reconstruction System, Phoenix 21216-10, Autal, Austria) buttressing the medial acetabular wall and the quadrilateral acetabular plate either using 3.5 mm conventional screws (Group A) or 4.2 mm locking screws (Group B) (Figure 1). Due to the superior strength/stability of the material of the screws (TiALV) compared with the material of the plate (titanium grade 2) the locking screw head forms a thread in the plate. The PRS Pelvis Reconstruction System has 15° off-axis variable angle screws. The anatomical pre-shaped plates were additionally pre-contoured manually to the shape of the bone to ensure optimal implant fit. The position of the plate was marked on each of the 16 pelvises for standardized and exact implant positioning [7]. In both groups, the 10-hole plate was fixed with either 6 conventional screws or 6 locking screws. 2 screws were inserted in the symphyseal holes of the plate and 1 screw was inserted in the superior pubic ramus [8]. Additionally, 1 posterior screw and 2 periarticular screws were inserted [9]. The use of periarticular screws in acetabular fractures involving the quadrilateral plate increases the overall stability significantly [9]. Optical markers were attached to the fracture sites for motion tracking.

### Biomechanical testing

**Figure 2:**
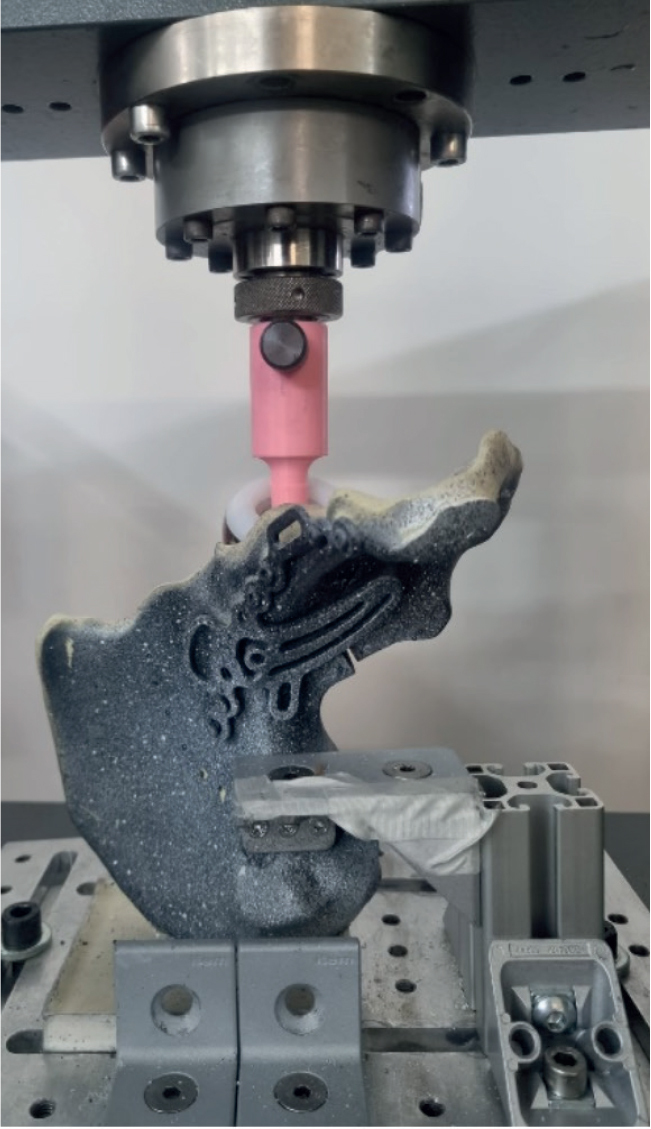
Force was applied in a medio-superior direction through the hemiarthroplasty using a previously described test set-up [20-24].

Biomechanical testing was performed with the use of an electrodynamic test system (Zwick Z005, Ulm, Germany) equipped with a 10 kN load cell (Figure 2). A previously described standardization of the acetabulum loading mechanism was performed using a unipolar hemiarthroplasty on the left acetabulum (Figure 2) [10-14]. The force was applied in a medio-superior direction through the hemiarthroplasty. The ilium was stabilized in a customized jig [10] and the pubic symphysis was allowed to freely rotate. Starting from 100 N the peak load of each cycle was increased at a rate of 0.63 N/cycle.

### Data acquisition

Interfragmentary displacements were measured in all 6 degrees of freedom by motion tracking (ARAMIS SRX, GOM GmbH, Braunschweig, Germany) at a rate of 10 Hz. The sensitivity of measurement was 0.004 mm in the XY plane (frontal to the cameras) and along the z-axis (depth) [7,15,16].

**Figure 3:**
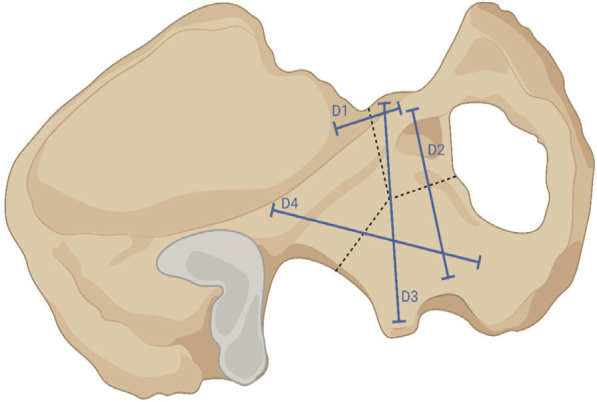
Interfragmentary displacement along the axes D1–D4 was measured by motion tracking. The dashed lines show the T-shaped acetabular fracture. This figure was created with BioRender (https://www.biorender.com/).

The primary outcome measure was displacement at the fracture sites and along the lines D1–D4 (Figure 3). D1 is the distance between the superior pubic ramus and the os ilium at the fracture site. D2 measures the distance between the superior pubic ramus and the os ischium at the fracture site. D3 presents the distance of os ilium and os ischium along the axis of load application. D4 is the distance between the os ilium and os ischium. The secondary outcome measure was the construct stiffness (N/mm). Construct stiffness was defined as force per axial displacement and was calculated from the force–displacement curve during initial loading with 100 N.

### Statistics

Statistical analysis was performed using IBM SPSS Statistics (v.27, IBM Corp, Armonk, NY, USA). The 2 data sets (Group A vs Group B) were considered independent. Descriptive data is presented as mean value with standard deviation (SD), and between-group differences as mean differences (∆) with 95% confidence intervals (CI). Normality of data distribution within each group was screened using the Shapiro–Wilk test, followed by the independent samples t-test to compare the normally distributed outcome measures. The Mann–Whitney U test was applied to compare the non-normally distributed outcome measures. Level of significance was set at 0.05.

An a priori power analysis was performed using G*Power-2 software (University of Düsseldorf, Düsseldorf, Germany) [17]. Based on the means and standard deviations from a previous study evaluating the biomechanical performance of infra-acetabular screw fixation in acetabulum fractures with posterior column involvement [13], it was assumed that a sample size of 8 in each group would allow the detection of changes in displacement of 0.2 mm with 95% power at the significance level of P < 0.05.

### Ethics, registration, funding, reporting guideline, and disclosures

Due to the study design no approval by an ethics committee was necessary. No funding was received. The study is reported according to the guidelines for running biomechanic studies [18]. The authors have no conflicts of interest to declare. Complete disclosure of interest forms according to ICMJE are available on the article page, doi: 10.2340/17453674.2024.42490

## Results

Due to the detailed and extensive pre-testing, all experiments for this study went well and could be included in the analysis. Displacement at all measured fracture sits was less in the locking plate fixation group.

### Displacement of D1

**Figure 4:**
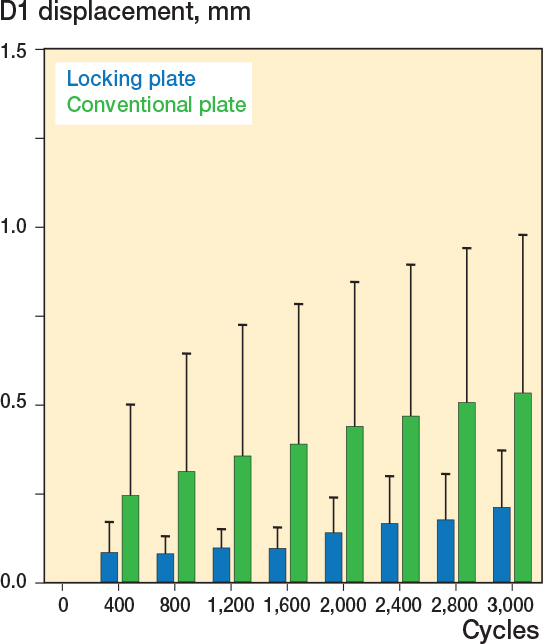
Fracture displacement along the D1 axis was less in the locking plate group.

Displacement between the superior pubic ramus and the os ilium (D1) was less in the locking plate fixation group. After 400 cycles fracture dislocation was higher in the conventional plate fixation group (0.18 mm) compared with the locking plate fixation group (0.06 mm, ∆ 0.11 mm, CI 0.03–0.26, Figure 4). After 1,600 cycles fracture dislocation was less in the locking plate fixation group (0.10 mm) compared with the conventional plate fixation group (0.28 mm, ∆ 0.17 mm, CI 0.02–0.37). After 3,000 cycles fracture dislocation was 0.40 mm in the conventional plate fixation group and 0.22 mm in the locking plate fixation group, demonstrating a difference between the 2 groups of 0.19 mm (CI 0.02–0.44).

### Displacement of D2

**Figure 5:**
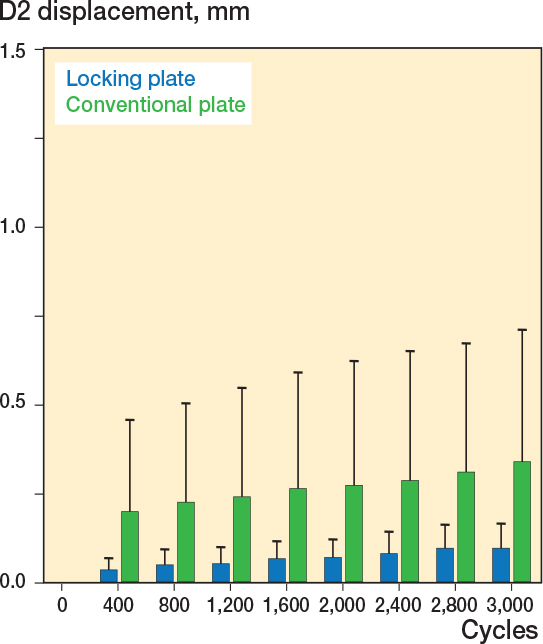
Fracture displacement along the D2 axis was less in the locking plate group.

Under cyclic loading displacement of the superior pubic ramus and os ischium (D2) was higher in the group of specimens with conventional plates. After 400 cycles fracture dislocation was higher in the conventional plate group (0.12 mm) compared with 0.04 mm in the locking plate fixation group (∆ 0.08 mm, CI 0.01–0.15, Figure 5). After 1,600 cycles fracture dislocation was not different in conventional plate fixation the group (0.16 mm) compared with the locking plate fixation group (0.07 mm, ∆ 0.09 mm, CI –0.01 to 0.19). After 3,000 cycles fracture dislocation was less in the locking plate fixation group compared with the conventional plate fixation group (0.10 mm vs 0.22 mm, ∆ 0.12 mm, CI 0.03–0.20).

### Displacement of D3

**Figure 6:**
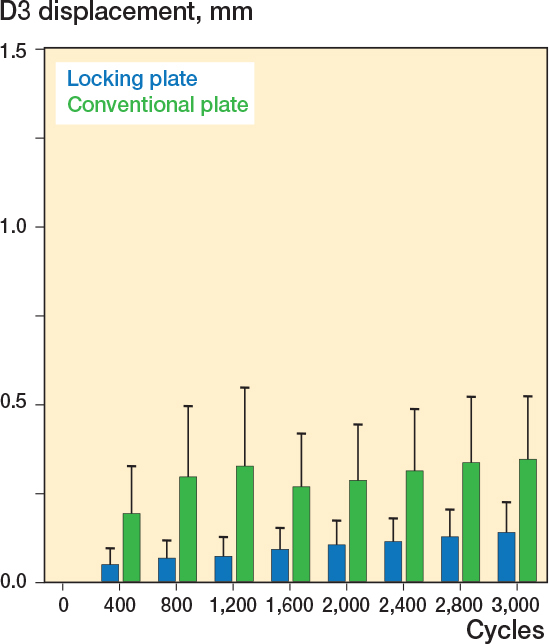
Fracture displacement along the D3 axis was less in the locking plate group.

Along the axis of load application displacement (D3) a difference between conventional plate fixation and locking plate fixation was detected for all measured cycles. After 400 cycles fracture dislocation was higher in the conventional plate group (0.17 mm) compared with the locking plate fixation group (0.05mm ∆ 0.12 mm, CI 0.01–0.23, Figure 6). After 1,600 cycles fracture dislocation was higher in the conventional plate group (0.25 mm) compared with the locking plate group (0.09 mm, ∆ 0.15 mm, CI 0.04–0.28). After 3,000 cycles fracture dislocation was less in the locking plate fixation group compared with the conventional plate fixation group (0.14 mm vs. 0.34 mm, ∆ 0.20 mm, CI 0.04–0.36).

### Displacement of D4

**Figure 7:**
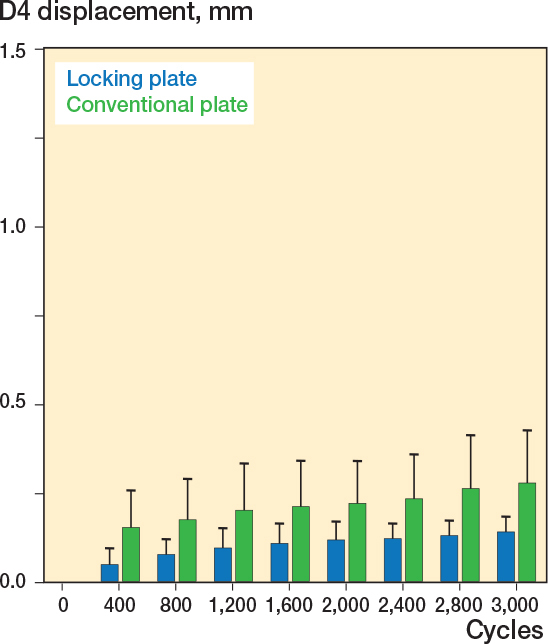
Fracture displacement along the D4 axis was less in the locking plate group.

The distance between os ilium and os ischium (D4) did not differ between the 2 groups. After 400 cycles no difference was detected between the conventional plate fixation group (0.14 mm) and the locking plate fixation group (0.05 mm, ∆ 0.08 mm, CI 0.00–0.17, Figure 7). After 1,600 cycles (0.19 mm in in the conventional plate fixation group vs.0.11 mm in the locking plate fixation group, ∆ 0.07 mm, CI –0.01 to 0.16) and after 3,000 cycles (0.26 mm in in the conventional plate fixation group vs 0.15 mm in the locking plate fixation group, ∆ 0.11 mm, CI –0.15 to 0.24) the fracture dislocation did not differ between the conventional plate fixation and locking plate fixation groups.

### Axial stiffness

**Figure 8:**
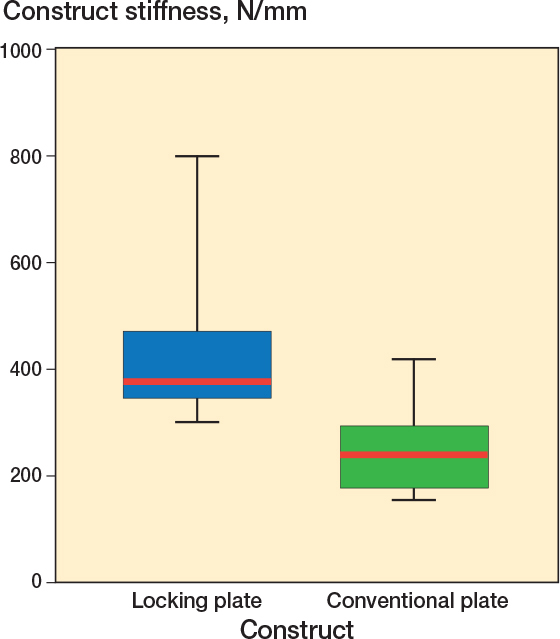
No statistically significant difference in initial axial stiffness was detected between locking plate and conventional plate osteosynthesis. Red lines are median construct stiffness. Boxes represent the interquartile ranges; the lower edge is the 1st quartile and the upper the 3rd quartile. The whiskers show the range of the data up to 1.5 times the interquartile range from the respective quartile without outliers.

No differences of mean initial axial stiffness were detected between angular stability (407 N/mm) and conventional plating (308 N/mm, ∆ 99 N/mm, CI –48 to 245, Figure 8).

## Discussion

The aim of the present study was to compare biomechanically conventional versus locking plate fixation in a T-shaped acetabular fracture. In brief, we showed that under an increasing cycling loading the angular stable locking implant shows less displacement.

Different biomechanical studies showed that fixation of both acetabular columns provides the greatest stability in complex fracture patterns [10]. Furthermore, the use of a quadrilateral surface buttress plate is comparable and in some biomechanical aspects is superior for fixation of acetabular fractures compared with traditional forms of transverse acetabulum fixation techniques [19]. Buttressing the medial acetabular wall led to more stability and a better outcome [2,8,11,13,16]. A further study observed that infra-acetabular screw placement is biomechanically advantageous [20]. Therefore, the present study used plates buttressing the quadrilateral plate only and infra-acetabular screw placement.

Culemann et al. compared biomechanically different stabilization techniques for acetabular fractures [8]. The locking reconstruction plate without buttress function was inferior compared with the buttress plate and the plate with the periarticular screws. Furthermore, the locked reconstruction plate without buttress function had no off-axis angle screw insertion option preventing periarticular screws, which is another major difference from the plate used in the present study. Interestingly, statistically significant results were seen only in the biomechanical tests with synthetic specimens and not in the cadaver specimens, due to inhomogeneous bone quality [8].

Another biomechanical study could not detect any superiority of a locking plate system compared with a non-locking plate system, which is not in accordance with the results of our study [20]. Again, the plates used did not buttress the quadrilateral surface of the acetabulum [20]. Mehin et al. [21] showed no significant difference in the fracture gap comparing conventional plate fixation without buttressing function and locking plate fixation without buttress function in transverse acetabular fractures in only 5 cadaver specimens with variable bone quality. However, a significant correlation between construct rigidity and fracture displacement in cyclic loading tests was observed, which is in accordance with the results of our study [21]. Also fracture gap after cyclic loading correlated significantly with the stiffness of the construct [21]. Due to the variable bone quality of the specimen used and the low number of specimens the study might be underpowered [21]. It is necessary to highlight that the previous biomechanical studies comparing locking and non-locking plates used implants not buttressing the quadrilateral surface of the acetabulum. Previous published studies showed the superiority of plates buttressing the medial acetabular wall. In our opinion, this major difference in implant design explains the different results.

A T-shaped acetabular fracture involving the quadrilateral plate is a complex fracture pattern and severe injury. This kind of fracture involves the critical weightbearing portion of the acetabulum. Due to the fracture of the quadrilateral plate (medial wall of the acetabulum), there is no bony structure to prevent medial subluxation of the femoral head. Typically, intrapelvic surgical approaches are used to address T-shaped acetabular fractures. Several surgical approaches and techniques have been described to address acetabular fractures with medial displacement patterns. Matta and Liebergall et al. have shown in their studies from the 1990s that open reduction and internal fixation using standard techniques led to less successful outcomes in the osteoporotic bone of elderly patients [22,23]. The description of the modified Stoppa approach by Cole and Bolhofner offering intrapelvic access and direct exposure to the quadrilateral plate was published in 1994 [24]. With the introduction of quadrilateral surface buttress plates, fracture patterns involving the medial wall can be fixed reliably and solidly [19]. Approaches that allow for direct visualization of the transverse fracture components of the anterior column, the posterior column, and the quadrilateral plate enable buttress plate fixation. Anatomic reduction and internal fixation of acetabular fractures involving the quadrilateral plate lead to good and moderate clinical functional outcome [2]. A large increase in acetabular fractures and surgical treatment in the elderly patient with reduced bone stock after low-impact trauma has been observed in recent years [1,2,25]. The inability of elderly patients to maintain postoperative weightbearing restrictions requires a surgical procedure which allows early full weightbearing mobilization [5,26]. A general contraindication for surgery of acetabular fractures is the risk of poor construct stability in patients with reduced bone stock [27]. Many studies have underlined the importance of optimal reduction to improve functional outcome after acetabular fractures [28,29]. In anterior column and both-column fractures, transtectal involvement with more than 2 mm displacement increases the load on the superior part of the acetabulum, which increases the risk of posttraumatic arthrosis [30]. Especially in cases of reduced bone quality it is mandatory to keep the anatomic reposition to reduce the risk of posttraumatic arthrosis [31,32]. Tannast et al. grade the reduction as anatomical (0–1 mm residual displacement), imperfect (2–3 mm residual displacement), or poor (>3 mm) [32]. Preservation of the intraoperatively achieved reduction is dictated by the stability of the fixation construct. In cases of fractures near to joints and cases of reduced bone quality, locking plate fixation can be biomechanically superior. From a clinical perspective, determining and comparing the cumulative survivorship of the hip after open reduction and internal fixation of displaced acetabular fractures using conventional or locking implants with a clinical follow-up of at least 2 years would be favorable [32]. Clinical studies examining the possible clinical effect and advantage of locking implants for treatment of complex acetabular fractures are pending.

### Limitations

The use of synthetic bone models is a limitation but eliminates the heterogeneity of bone quality and geometry as confounding factors [8,19]. This leads to standardization, overwhelming the almost uncountable variations in bone quality seen in human cadaveric specimen [33].

### Strengths

The different diameter of the conventional screws (3.5 mm) and locking screws (4.2 mm) used might have influenced the results. However, these screw types were used in accordance with the surgical technique instruction of the implants used. The plates providing the possibility of using conventional and locking screws had the same design, thus guaranteeing a high level of comparability. The use of screws with the exact screw diameter would be desirable for the outstanding clinical studies. Further advantages of the present study were the relatively high number of specimens tested and the continuous measurement of the fracture displacement in all 6 degrees of freedom using 2 optical cameras with a very precise motion tracking system.

### Conclusion

The results of this study show that locking plate fixation buttressing the medial acetabular wall is, in the present biomechanical set-up using synthetic bone models, superior compared with conventional plating with buttress function, but no change in stiffness was shown. Clinical studies examining the use and possible advantage of locking implants in complex acetabular fractures of the elderly are required.

## Notes

ML, CK, MK, OR: conceptualization, design, methodology. ML, MK: testing, data acquisition, statistical analysis. ML: writing original draft. CK, MK, CP, KA, AG, MR, OR: internal review. KA, AG: testing, data acquisition. ML, CK, MR, OR: project administration, supervision, validation.

Handling co-editors: Ivan Hvid and Robin Christensen

*Acta* thanks Michael Brix and Morten Schultz Larsen for help with peer review of this manuscript.
